# Cardiac and Renal Effects of Atrasentan in Combination with Enalapril and Paricalcitol in Uremic Rats

**DOI:** 10.1159/000355811

**Published:** 2014-09-19

**Authors:** Cynthia Ritter, Sarah Zhang, Jane L. Finch, Helen Liapis, Edu Suarez, Leon Ferder, James Delmez, Eduardo Slatopolsky

**Affiliations:** a Renal Division; b Department of Pathology & Immunology, Washington University School of Medicine, St. Louis, MO, USA; c Department of Physiology and Pharmacology, Ponce School of Medicine and Health Sciences; d Department of Biology, University of Puerto Rico in Ponce, Ponce, Puerto Rico

**Keywords:** ACE inhibitor, Endothelin-1 receptor A, Renal failure, Uremic rats, VDRA, Vitamin D

## Abstract

**Background/Aims:**

The search for new therapies providing cardiorenal protection in chronic kidney disease (CKD) has led to treatments that combine conventional renin-angiotensin-aldosterone-system inhibitors with other drugs that exhibit potential in disease management.

**Methods:**

In rats made uremic by renal ablation, we examined the effects of addition of the endothelin-A receptor antagonist atrasentan to a previously examined combination of enalapril (angiotensin converting enzyme inhibitor) and paricalcitol (vitamin D receptor activator) on cardiac and renal parameters. The effects of the individual and combined drugs were examined after a 3-month treatment.

**Results:**

A decrease in systolic blood pressure, serum creatinine and proteinuria, and improvement of renal histology in uremic rats were attributed to enalapril and/or paricalcitol treatment; atrasentan alone had no effect. In heart tissue, individual treatment with the drugs blunted the increase in cardiomyocyte size, and combined treatment additively decreased cardiomyocyte size to normal levels. Perivascular fbrosis was blunted in uremic control rats with atrasentan or enalapril treatment.

**Conclusions:**

We found distinct cardiac and renal effects of atrasentan. Combination treatment with atrasentan, enalapril and paricalcitol provided positive effects on cardiac remodeling in uremic rats, whereas combination treatment did not offer further protective effects on blood pressure, proteinuria or renal histology.

## Introduction

Cardiovascular disease (CVD), a frequent complication of renal disease, is the major cause of mortality and morbidity in end-stage renal failure [1]. The renin-angiotensin-aldosterone system (RAAS) regulates extracellular volume homeostasis, which contributes to blood pressure stability. In the kidney, continual over-activation of the RAAS results in glomerular hypertension, fibrosis, and proteinuria, and leads to progressive renal damage [2, 3]. Agents that inhibit RAAS, such as angiotensin converting enzyme inhibitors (ACEIs) and angiotensin II receptor blockers (ARBs) have beneficial effects in patients with both chronic renal and cardiovascular diseases [4, 5].

Calcitriol (1,25-dihydroxyvitamin D_3_) and its analogs have a therapeutic potential in attenuating experimentally-induced kidney diseases [6-11]. These studies show suppression of the progression of glomerulosclerosis and albuminuria, and a decrease in podocyte loss in subtotally nephrectomized rats following vitamin D treatment. In addition, clinical studies show that vitamin D and its analogs are associated with decreased morbidity and mortality in patients with CKD [12-14]. Vitamin D deficiency is associated with increasing levels of albuminuria and markers of renal inflammation in patients with kidney diseases [15]. Additionally, paricalcitol, an analog of calcitriol and a vitamin D receptor activator (VDRA), decreases markers of renal inflammation in this patient population [16] and decreases proteinuria in patients with type 2 diabetic nephropathy [17]. Furthermore, our group has recently demonstrated that combination therapy of paricalcitol and enalapril (an ACEI) has anti-inflammatory and anti-oxidative stress pathways in the uremic rat model [7, 18], and has examined the molecular mechanisms underlying these properties [19].

Endothelial cells modulate vascular tone by releasing the vasodilation factor nitric oxide and the potent vasoconstrictor protein endothelin-1, the proinflammatory, mitogenic, and fibrotic effects of which contribute to the progression of renal failure [20]. Endothelin-1 binds to two different receptors, endothelin receptor A (ET_A_) and endothelin receptor B (ET_B_). ET_A_ receptors have vasoconstrictor and growth-promoting functions, whereas ET_B_ receptors mediate vasodilation and inhibition of growth and inflammation via release of nitric oxide. Atrasentan is a highly selective endothelial receptor agonist (ET_A_R) with ∼1800-fold greater affinity for the ET_A_ receptor than the ET_B_ receptor. A recent clinical trial demonstrated that atrasentan exhibited additive effects in decreasing the urine albumin-to-creatinine ratio in patients with type 2 diabetic nephropathy who were receiving ACEIs or ARBs [21].

The aim of this study is to investigate the effect of atrasentan, enalapril and paricalcitol, individually and in combination, on cardiac and renal histology and function in the 5/6 nephrectomized uremic rat model.

## Materials and Methods

### Experimental Protocol

The Animal Studies Committee at Washington University School of Medicine approved all experimental protocols in accordance with federal regulations. Uremia was induced in a group of female Sprague-Dawley rats (225-250g; approximately 8 weeks old) by 5/6 nephrectomy. Nephrectomized rats were divided into 5 groups and preventive treatment started 1 day after surgery as follows: uremic control (UC); uremic + enalapril (UE, 20 mg/kg in drinking water); uremic + atrasentan (UA, 10 mg/kg/day, in drinking water); uremic + paricalcitol (UP, 200 ng/rat administered IP three times per week); and uremic + enalapril + paricalcitol + atrasentan (UEAP). Normal rats served as control (NC). Doses of enalapril and paricalcitol were chosen based on our previous studies [7, 19]. The dose for atrasentan was chosen based on a study by Saleh, et al. and via personal communication with one of the authors [22], and is within the range of atrasentan doses used in recent studies (3-10 mg/kg/day) [23-26]. All rats were fed normal rat chow containing 0.9% calcium, 0.4% phosphorus, and 0.259% sodium chloride. Rats were treated for 3 months and then killed by exsanguination via the dorsal aorta and blood was taken for determination of serum chemistries. Blood pressure was determined using the tail cuff method using the Non-Invasive BP System XBP1000 (Kent Scientific, Torrington, CT) before surgery, at 1, 2, and 3 months (just prior to sacrifice). Samples of blood and urine were taken before surgery and at 1 and 2 months. Just prior to sacrifice rats were placed in metabolic cages and 2-24 hour urine collections were made. Serum creatinine, calcium and phosphorus, and urinary albumin were determined as previously described [27]; results reflect the average of values obtained from the 2 urine collections. The remnant kidney was divided into sections. One section was fixed in 10% buffered formalin for histological examination and the rest were snap-frozen in liquid nitrogen and stored at -80°C. The entire myocardium was removed and left ventricle dissected away and weighed. Sections of left ventricle were fixed in 10% buffered formalin for histological examination.

### Analytical determinations

Serum P and creatinine were measured by an autoanalyzer (COBAS-MIRA Plus, Branchburg, NJ, USA). Total serum calcium was measured by atomic absorption spectrophotometry (Perkin-Elmer, model 1100B, Norwalk, CT, USA). Ionized Ca was measured using a Nova 8 electrolyte analyzer (Nova Biomedical, Woltham, MA, USA).

### Histochemistry and assessment of glomerulosclerosis and interstitial infiltration

Formalin-fixed, paraffin sections of kidney tissue were stained with Hematoxylin/Eosin and Masson's Trichrome to assess glomerulosclerosis, interstitial inflammation, interstitial fibrosis, tubular atrophy, tubular dilation and calcifications as previously described [19]. Each histopathological feature was scored 1-4 as follows: 1≤10% of surface area involved; 2=10-24%; 3=25-50% and 4≥50%.

### Immunohistochemical evaluation of inflammation in kidney

Immunohistochemical staining for CD-68 (Cluster of Differentiation 68), a marker of monocyte/macrophage cells was performed as previously described [7], with the exception that 2.5% horse serum was used for blocking, and mouse Impress™ as the second antibody (Vector Laboratories; Burlingame, CA). The CD-68-positive cells were counted manually in 10 cortical areas including glomeruli (400×) and expressed as number of positive cells per high powered field. Results are reported as mean ± s.e.m; N=6 for N, UC, UP UEAP groups; n=5 for UE and UA groups.

### Real-time PCR for markers of inflammation in the kidney

Kidney samples were analyzed by real-time (RT) quantitative PCR. Total RNA was isolated using RNAzol Bee (Tel-Test, Friendswood, TX). Reverse transcription of the RNA was carried out using oligo-dT primer and SMART MMLV reverse transcriptase (CLONTECH Laboratories, Mountain View, CA). RT-PCR was performed using Fast SYBR Green Master Mix (Applied Biosystems; Foster City, CA) in an Applied Biosystems 7900HT Fast Real-Time PCR System. QuantiTect Primer Assays obtained from Qiagen (Valencia, CA) were obtained for quantifying rat transforming growth factor-beta 1 (TGF-β1; QT00187796), the chemokine monocyte chemoattractant protein (MCP-1; QT00183253),), nuclear factor kappa-b subunit 1 (NFk-B1; QT00370545) and the housekeeping gene glyceraldehyde 3-phosphate dehydrogenase (GAPDH). The ΔΔC^T^ technique was used to calculate the ratio of target gene to GAPDH, with respect to normal control rats. Results were reported as percent of normal control values, which represented 100%.

### Myocardial Histology

Cross-sectional cuts of formalin-fixed, paraffin-embedded left ventricle were stained with Masson's Trichrome and analyzed using Image-Pro Plus software, Version 7.0 (Media Cybernetics, Silver Spring, MD). Perivascular fibrosis was assessed by calculating the percentage of Trichrome-stained collagen deposits surrounding the vessel to the total perivascular area using the software's color cube function. Approximately 10 vessels were assayed for each rat (n=6 rats/group). The area of individually circumscribed cardiomyocytes was determined using the ImagePro software. The average cardiomyocyte size (pixels) from ∼100 cells/rat was expressed as unit area.

### Statistical analysis

All data are expressed as mean SEM. One-way analysis of variance (ANOVA) with the Tukey's post-test, unless stated otherwise, was used for comparison between uremic groups. Student's nonpaired *t*-test was used to compare the normal control groups with individual uremic groups or when only two groups were compared. The Kaplan-Meier method was used to construct survival curves, and a log-rank (Mantel-Cox) test was used to analyze differences between the groups (GraphPad Prism 6.0; GraphPad Software; La Jolla, CA). P ≤ 0.05 was considered significant.

## Results

### Serum chemistries

Results from serum chemistries are shown in Table 1. As expected, serum creatinine (Cr) was significantly increased in all uremic groups compared to normal. Treatment with enalapril (UE and UEAP) significantly blunted this increase. Body weight did not change in any of the groups. A significant increase was observed in ICa^++^ with paricalcitol treatment (5.46 ± 0.07 for UP and 5.29 ± 0.06 mg/dl for UEAP) compared to normals (4.84 ± 0.04 mg/dl) and uremic controls (4.90 ± 0.04 mg/dl). Serum phosphorus and CaxP product were significantly elevated in all uremic rats, except for those treated with enalapril alone.

### Urinary findings

**Fig. 1:**
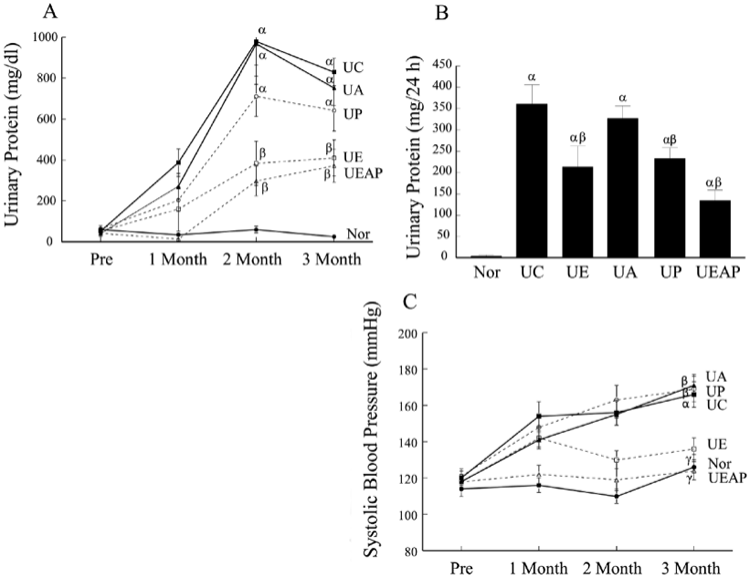
A) Spot-urine protein concentration time course for pre-surgery, 1, 2, and 3 months after surgery for uremic rats receiving no treatment (UC; n=13), or treatment with enalapril (UE; n=13), atrasentan (UA; n=11), paricalcitol (UP; n=13), or a combination of the 3 drugs (UEAP; n=13). Normal rats served as controls (Nor; n=6). Results are expressed as mean ± SEM; ^α^p≤0.05 vs. Nor, ^β^p≤0.05 vs. UC. B) 24-hour urinary protein excretion at 3-months. ^α^p≤0.05 vs. Nor; ^β^p≤0.05 vs. UC; ^γ^p<0.05 UP vs. UC with T-Test. C) Systolic blood pressure. Blood pressure was measured monthly for 3 months. ^α^p<0.0001 UC baseline pre-treatment vs. UC 3-month time point; ^β^p<0.01 UA and UP vs. UC 3-month time point; ^γ^p<0.01 UE and UEAP vs. UC 3-month time point.

A time-course for urinary protein levels of spot-urine samples collected pre-surgery and at 1, 2, and 3 months of the study is shown in figure 1A; these values follow the same pattern as for a 24-hour urinary protein collected at the end of the study (3 months), as shown in figure 1B. Uremia resulted in a significant rise in 24 hr protein excretion, from 4.4±1.8 mg/24 hr in normal rats (n=6) to 360.6±45.4 in uremic controls (n=13). Proteinuria was markedly decreased in rats treated with enalapril (213.2 ± 48.7 mg/24 h, n=13; p≤0.05 vs. UC) or paricalcitol (233.5±24.6, n=13; p≤0.05 vs. UC). The combination treatment (UEAP) resulted in an even more marked reduction (134.7±24.1 mg/24 hr; p≤0.05 vs. UC). Treatment with atrasentan had no effect on proteinuria (305±34.3).

### Blood pressure

Systolic blood pressure was measured throughout the study and is shown in figure 1C. As expected, blood pressure in untreated uremic rats (uremic controls) increased over 3-months, from a baseline measurement of 120±4 to 166±7 mm Hg after 3 months of uremia (p<0.01). Three months of treatment with atrasentan or paricalcitol had no effect on the increase in systolic blood pressure (169±7 and 171±6 mm Hg, respectively). As expected, enalapril prevented this increase in blood pressure (136±6 mm Hg, p<0.01 vs. UC). Combination treatment normalized blood pressure (124±5 mm HG, p<0.01 vs. UC).

### Survival

**Fig. 2:**
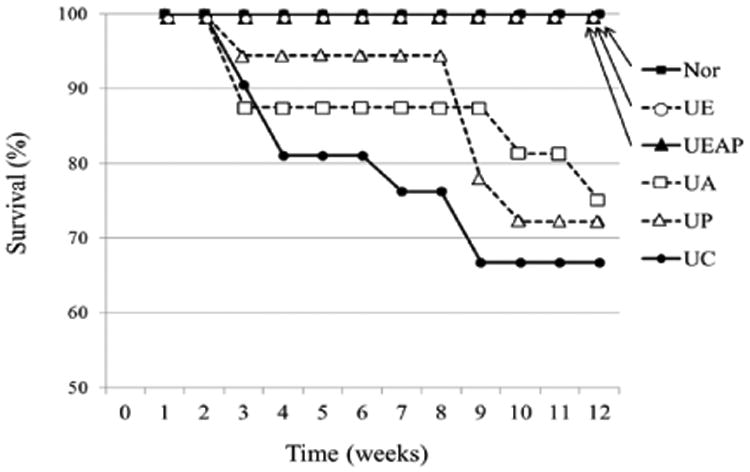
Survival rates over 3 months in uremic rats receiving no treatment (UC), or treatment with enalapril (UE), atrasentan (UA), paricalcitol (UP), or a combination of the 3 drugs (UEAP). Normal rats served as controls (Nor).

Kaplan-Meier survival curves are shown in figure 2. No rats died in the normal control group (n=6), or in groups receiving either enalapril alone (UE; n=15) or in combination treatment (UEAP; n=13). Uremic control rats (UC) had a mortality rate of 33.3% (7 of 21 rats died; Mantel-Cox long rank test, p= 0.068). Mortality was 27.8% (5 of 18) for paricalcitol treatment and 25% (4 of 16) for atrasentan treatment.

### Assessment of kidney histology, interstitial infiltration, and markers of inflammation and fibrosis

**Fig. 3:**
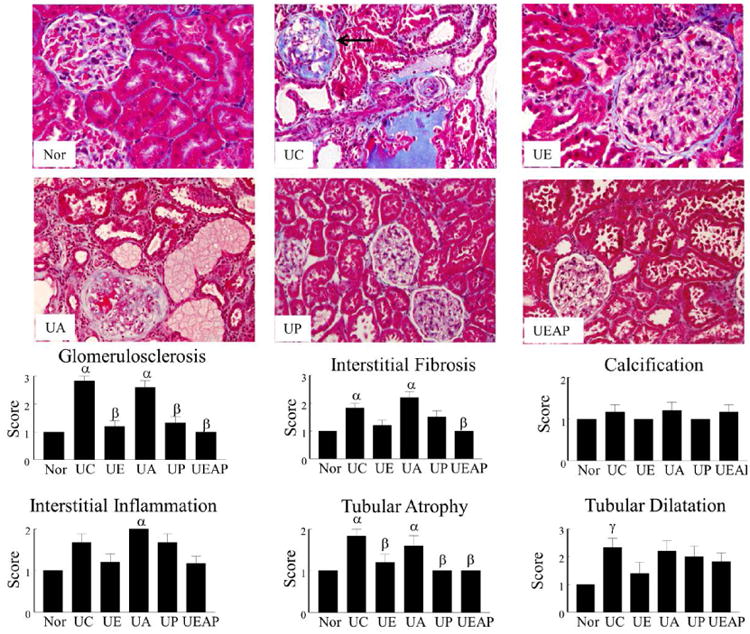
Upper panel: Representative images of renal histology in uremic rats receiving no treatment (UC; arrow indicates glomerulosclerosis), or treatment with enalapril (UE), atrasentan (UA), paricalcitol (UP), or a combination of the 3 drugs (UEAP). Normal rats served as controls (Nor). Trichrome stain, 200×. Lower panel: Quantitation of renal histology. Scoring is as follows: 1 = <10%, 2 = 11-24%, 3 = 25-50%. ^α^p ≤ 0.01 vs Nor, ^β^p ≤ 0.01 vs UC, ^γ^p ≤ 0.05 vs. Nor.; n=6 each.

Figure 3 shows representative images of kidney sections stained with Masson's Trichrome (upper panel), and quantitation of histological changes (lower panel). Histology of normal kidneys (Nor) showed intact glomeruli and no evidence of interstitial fibrosis. Uremic control kidneys (UC) showed markedly increased interstitial fibrosis and inflammation, glomerulosclerosis (arrow), tubular dilatation and atrophy. Uremic rats treated with enalapril (UE) showed absent glomerulosclerosis, and minimal interstitial fibrosis and tubular atrophy. Treatment with atrasentan (UA) had no effect on glomerulosclerosis, interstitial fibrosis, inflammation, tubular dilatation or atrophy. Treatment with paricalcitol (UP) showed minimal interstitial fibrosis and no tubular atrophy or glomerulosclerosis. Combination treatment (UEAP) resulted in the greatest protective effect on renal histology, with the kidney exhibiting no glomerulosclerosis, interstitial fibrosis, interstitial inflammation, or tubular atrophy, and only mild tubular dilatation. No appreciable calcification was found in any group.

**Fig. 4:**
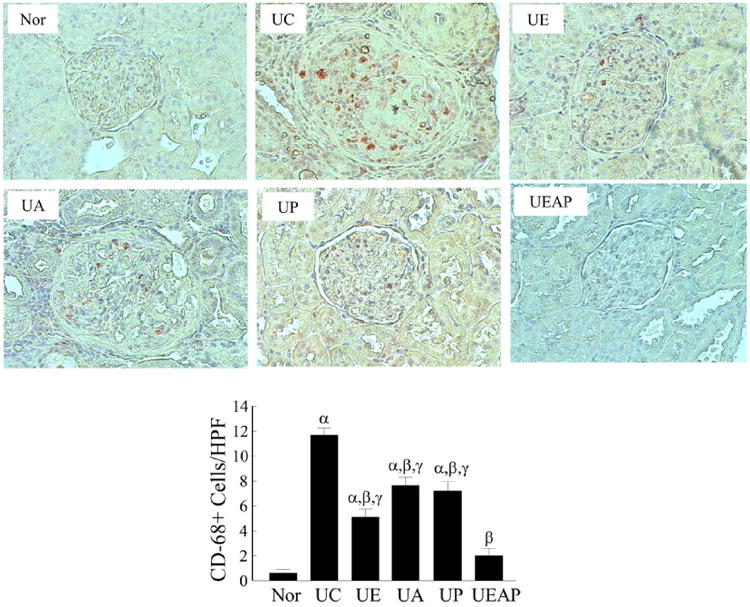
Upper panel: Representative images of CD-68 stained sections of kidneys from normal rats (Nor), and kidneys of uremic rats receiving no treatment (UC), or treatment with enalapril (UE), atrasentan (UA), paricalcitol (UP), or a combination of the 3 drugs (UEAP); 400×. Lower panel: Quantitation of CD-68-positive cells in kidneys of Nor (n=6), UC (n=6), UE (n=5), UA (n=5), UP (n=6), and UEAP (n=6). ^α^p ≤ 0.001 vs Nor, ^β^p ≤ 0.001 vs UC, ^γ^p ≤ 0.05 vs. UEAP.

Macrophage infiltration in the kidney was assessed by immunohistochemistry for the macrophage/monocyte marker CD-68. Representative images of the staining and quantitation are shown in figure 4, upper and lower panels, respectively. CD-68 expression in normal kidney (0.63 ± 0.3 positive cells per high powered field; HPF) increased 18-fold in uremic control rats (11.7 ± 0.59, p<0.001). All 3 drugs, individually, significantly blunted this increase (enalapril: 5.12 ± 0.65; atrasentan: 7.64 ± 0.68; and paricalcitol: 7.23 ± 0.72 positive cells/HPF, p<0.001 for each). The combination treatment produced the most dramatic reduction in CD-68 expression (2.01 ± 0.59 positive cells/HPF, p<0.001 vs. UC), which was significantly lower than each drug alone, indicating an additive effect of combined treatment.

**Fig. 5:**
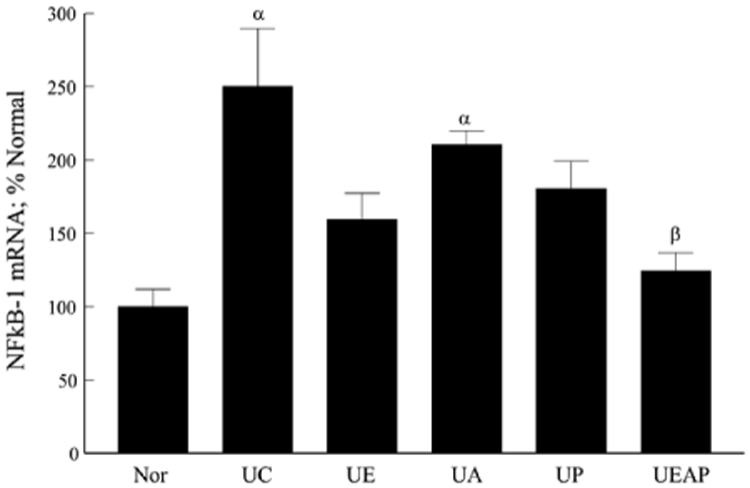
Quantitation of renal NFkB-1 mRNA. Data are expressed as the ratio of target mRNA to GAPDH mRNA (ΔΔC^T^ method). ^α^p ≤ 0.05 vs. Nor.; ^β^p ≤ 0.05 vs UC; N=4 each.

**Fig. 6:**
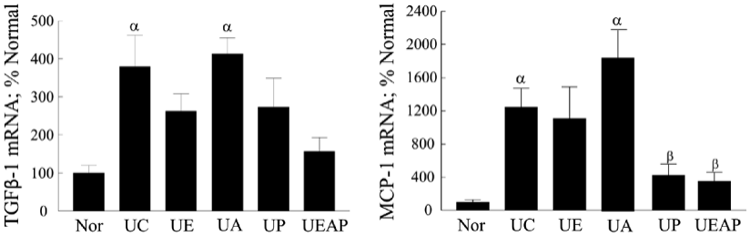
Left panel: Quantitation of renal TGFβ-1 mRNA. Data are expressed as the ratio of target mRNA to GAPDH mRNA (ΔΔC^T^ method). ^α^p ≤ 0.05 vs. Nor.; N=6 each. Right panel: Quantitation of renal MCP-1 mRNA Data are expressed as the ratio of target mRNA to GAPDH mRNA (ΔΔC^T^ method). ^α^p ≤ 0.05 vs. Nor.; ^β^p ≤ 0.05 vs UC; N=6 each.

Expression of NFkB-1 mRNA in the uremic control kidney was significantly increased 2.5-fold compared to the normal kidney (p≤0.05) as shown in figure 5. Treatment with enalapril and paricalcitol blunted the increase in NFkB-1 (1.6- and 1.8-fold increase) and the combination treatment group expressed the lowest levels compared to normal rats (1.2-fold increase; p≤0.05 versus uremic controls). Atrasentan had no effect on NFkB-1 expression. The expression of TGFβ-1 mRNA was similar to that of NFkB-1. Expression of TGFβ-1 mRNA in the uremic control kidney was significantly increased 3.8-fold compared to normal kidneys (p≤0.05; figure 6-left panel). Treatment with enalapril and paricalcitol blunted the increase in TGF-β (2.6- and 2.7-fold increase), and the combination treatment group expressed the lowest levels compared to normal rats (1.6-fold increase). Atrasentan had no effect on TGFβ-1 expression. Expression of MCP-1 mRNA in the uremic control kidney was significantly increased 12.5-fold compared to normal kidneys (p≤0.05; figure 6-right panel). Treatment with paricalcitol blunted the increase in MCP-1 (4.3-fold increase), whereas enalapril and atrasentan did not (11.1-fold and 18.4-fold increase). The combination treatment also blunted the increase in MCP-1 mRNA (3.6-fold increase compared to normal rats), with values significantly decreased compared to the uremic controls (p≤0.01).

### Myocardial Histology

**Fig. 7:**
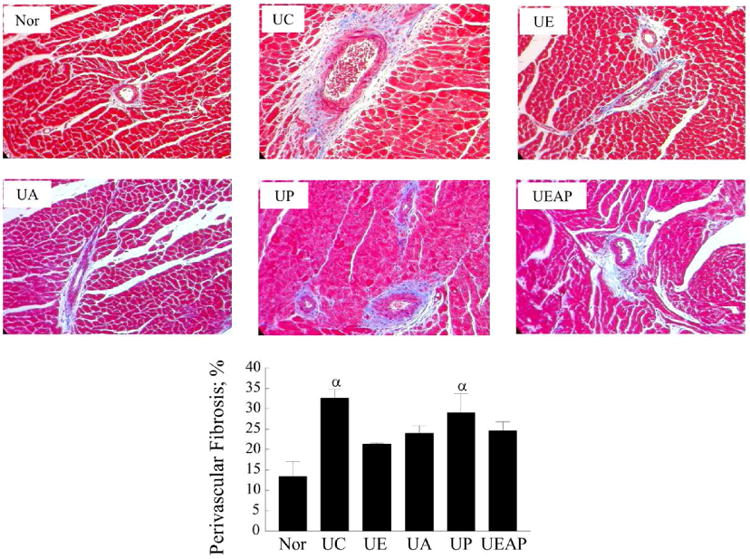
Representative images (top panel) and quantitation (bottom panel) of perivascular fibrosis in left ventricles of uremic rats receiving no treatment (UC; n=6), or treatment with enalapril (UE; n=6), atrasentan (UA; n=6), paricalcitol (UP; n=6), or a combination of the 3 drugs (UEAP; n=5). Normal rats served as controls (Nor; n=6). ^α^p ≤ 0.01 vs Nor. 200× magnification.

Perivascular fibrosis in the left ventricle was assessed by deposition of collagen around the vasculature. Figure 7 shows representative images of collagen deposition (blue color, top panel) and quantitation of fibrosis (bottom panel). Perivascular fibrosis was significantly higher in untreated uremic rats compared to normal controls (32.7 ± 2.0 vs.13.4 ± 3.6%, p≤0.01). This increase in fibrosis in uremic rats was blunted with treatment with enalapril (21.3 ± 0.3%) or atrasentan (24.0 ± 1.8%). Combination treatment did not augment this effect (24.6 ± 2.2%). Paricalcitol alone (29.01 ± 4.65%) had little effect.

**Fig. 8:**
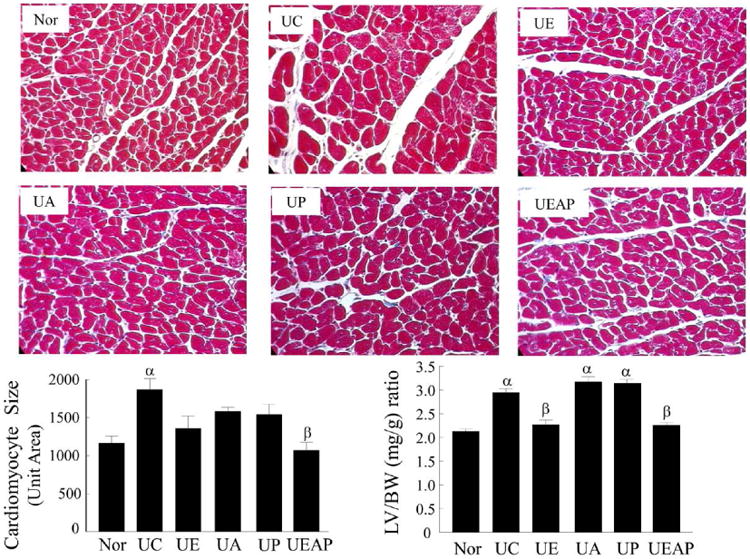
Representative images (top panel, 400× magnification) and quantitation of cardiomyocyte size in left ventricles (bottom left panel) are shown for uremic rats receiving no treatment (UC; n=6), or treatment with enalapril (UE; n=6), atrasentan (UA; n=5), paricalcitol (UP; n=4), or a combination of the 3 drugs (UEAP; n=6). Normal rats served as controls (Nor; n=5). ^α^p ≤ 0.01 vs Nor, ^β^p ≤ 0.01 vs UC. Left ventricular weight (bottom right panel) is corrected for body weight and reported as the ratio of LV/BW (Nor=6, UC=13, UE=14, UA=11, UP=13, and UEAP=13). ^α^p ≤ 0.001 vs Nor, ^β^p ≤ 0.001 vs UC.

Figure 8 shows representation images of left ventricular cardiomyocytes (top panel), quantitation of cardiomyocyte size (bottom left panel) and left ventricular weight (bottom right panel). Cardiomyocyte size was significantly increased in untreated uremic rats compared to normal controls (1867 ± 139 vs.1163 ± 92 unit area, p≤0.01). While the individual treatments of enalapril (1359 ± 163), atrasentan (1580 ± 55) or paricalcitol (1538 ± 137) blunted this increase, an additive effect of combined treatment significantly decreased cardiomyocyte size to normal levels (1067 ± 105, p≤0.01 vs. UC). Only enalapril, alone or in the combination group, blunted left ventricular weight.

## Discussion

Despite recent advances in CKD treatment, progression to end-stage renal disease and the development of cardiovascular events are still major problems for many CKD patients. The search for new therapies that will prevent, or even reverse, the progression of kidney disease has led to treatments that combine conventional RAAS inhibitors with other drugs that have shown promise for cardiorenal protection in CKD patients. Recent work from our group has demonstrated that combination therapy of paricalcitol and enalapril improved renal function and histology, suppressing the progression of renal failure via mediation/inhibition of the TGFβ-1 signaling pathway [7, 18, 19]. The present study is an extension of our previous works and seeks to answer the question of whether addition of an ET_A_R to a treatment regimen consisting of an ACE inhibitor and VDRA would further improve cardiac and renal functions in the uremic rat model.

Since chronic activation of the endothelin system in CKD contributes to the rate of progression of the disease, targeted blocking of this system may aid in the management of kidney disease. Highly specific ET_A_Rs (atrasentan, sitaxsentan) have been investigated for their protective effects on cardiac and renal systems. Here, we found that atrasentan had distinct effects in the heart and kidney. Regarding cardiac remodeling, individual treatment with atrasentan, enalapril or paricalcitol blunted the increase in cardiomyocyte size, and combined treatment significantly decreased cardiomyocyte size to levels comparable to controls. The finding that cardiomyocyte size, but not LV weight, was blunted by atrasentan and paricalcitol may be attributed to changes in LV mass resulting from not only cardiomyocyte size, but also an increase in non-cardiomyocytes (i.e., fibroblasts) or extracellular matrix. We previously showed that proliferation of cardiac interstitial cells dramatically increased in uremic animals [28]. In the present study, it is possible that atrasentan and paricalcitol blunted an increase in cardiomyocyte size, but were unable to affect the accumulation of interstitial cells or matrix. Further analysis of this theory is warranted. Perivascular fibrosis was blunted in uremic rats with atrasentan or enalapril treatment, but combination treatment showed no additive effect. The lack of an effect of paricalcitol on LV perivascular fibrosis was unexpected, since we previously showed that paricalcitol ameliorated interstitial and perivascular fibrosis in uremic rats (although this was only observational) [29]. However, Panizo et al. [30] recently showed that paricalcitol was effective in reducing myocardial fibrosis in uremic rats.

The inflammatory process resulting in renal fibrosis is complex, involving a multitude of cytokines/chemokines, growth factors, adhesion molecules and signaling pathways [31]. Renal interstitial infiltration of inflammatory cells includes neutrophils, macrophages, lymphocytes, dentritic cells and mast cells. Macrophages are major producers of TGFβ-1, which is crucial to the development of renal fibrosis. TGFβ-1 itself is a potent chemoattractant for monocyte/macrophages, as is MCP-1 [32]. Additionally, the transcription factor NFkB-1 is important for the onset of inflammation through activation of specific genes, including MCP-1. Here, individual treatment with atrasentan, enalapril or paricalcitol reduced monocyte/macrophage infiltration. Combination treatment further suppressed levels of infiltration to within normal limits, suggesting an additive effect of the drugs. Enalapril and paricalcitol blunted the increase in expressions of TGFβ-1, NFkB-1 and MCP-1. Combination treatment provided the most significant suppression of these markers of inflammation and fibrosis, but was due to the combination of enalapril and paricalcitol since atrasentan alone had no effect. Similarly, the beneficial effects of combination treatment on renal histology were attributed to the effects of enalapril and/or paricalcitol since atrasentan alone had no effect. The importance of the finding that atrasentan decreased the number of CD-68-positive cells in the kidney is minimized by its lack of effect on interstitial inflammation or other histological aspects of tubular interstitial damage, as well as its lack of effect on TGFβ-1, NFkB-1 and MCP-1.

Atrasentan had a very modest (though non-significant) decrease in mortality and no effect on proteinuria, blood pressure, or kidney histopathology. The lack of an effect of atrasentan on proteinuria is in contrast to recent clinical trials. In patients with type-2 diabetic nephropathy receiving ACEIs and/or ARBs, treatment with atrasentan produced an additive effect in decreasing the urine albumin-to-creatinine ratio [21]. In another study, CKD patients receiving ACEIs and/or ARBs plus sitaxsentan had reduced proteinuria and blood pressure and improved arterial stiffness [33]. Experiments in animals using monotherapy or combination therapy with ET_A_Rs, however, have produced variable results. In one study using streptozotocin-induced diabetic mice, atrasentan reduced diabetic urine flow, proteinuria and plasma creatinine levels, and positively affected myocardial dysfunction [24]. In experimental type-1 diabetic rats, atrasentan[23, 24] was as effective as the ACEI ramipril in reducing cardiac fibrosis and improving systolic and diastolic function during reperfusion studies [23]. Combining the two treatments did not produce additive effects, possibly due to a maximal effective dose for each drug being used. In another study, uremic Ren-2 transgenic rats (hypertension model) were treated with an ACEI, atrasentan, or a combination. Atrasentan improved the survival rate, reduced blood pressure, attenuated the development of cardiac hypertrophy, and transiently reduced proteinuria. Combination therapy did not provide additive protection [25].

More consistent with our findings were the results of a study in rats with type-2 diabetes, in which cardiorenal effects were examined after treatment with sitaxsentan, ramipril, or combination treatment [34]. Ramipril ameliorated proteinuria and glomerulosclerosis; sitaxsentan did not. While ramipril or sitaxsentan reduced accumulation of renal monocyte/macrophages, combination therapy further reduced accumulation of monocytes/macrophages to normal levels. Combination therapy also reversed cardiomyocyte hypertrophy in the diabetic rats. Additionally, sitaxsentan treatment (more than ramipril) restored heart capillary density to levels comparable to controls. This protective effect of sitaxsentan on cardiac structure/architecture was due to the action of VEGF, a major mediator of neovascularization in the heart. Similar findings were reported in diabetic rats [35]. Moreover, beneficial cardiovascular effects of atrasentan are shown in non-kidney-related diseases, such as patients with early atherosclerosis [36, 37].

## Conclusion

In the present study, atrasentan improved cardiac fibrosis, yet had no effect on renal fibrosis. We speculate that this difference may be due to the nature of damage inflicted upon the respective tissues. The method used for inducing uremia is an immediate, harsh insult to the renal tissue, whereas the effect on cardiac tissue is a consequence of the subsequent kidney damage. An immediate vs. delayed damage to renal and cardiac tissue, respectively, as well as possible differences in the pathways that lead to fibrosis in each tissue may have resulted in differential effects of atrasentan. Further studies are necessary to clarify this issue. Our study has several limitations. First, since it is a preventative study, it is difficult to translate our findings into a setting with patients who arrive for treatment after damage has already taken place. Second, a dose response of the effect of atrasentan on study parameters should have been performed before deciding on a final dose of the drug for the study. Third, a control group of enalapril and paricalcitol combination treatment should have been included.

Clearly, there are distinct cardiac and renal effects of atrasentan and other ET_A_Rs. We found that protective effects on blood pressure, proteinuria and renal histology were due to enalapril and/or paricalcitol treatment, in agreement with previous reports. However, treatment with atrasentan alone or in combination with enalapril and paricalcitol provided positive effects on cardiac remodeling in uremic rats. An in-depth analysis of the effect of combination treatment of atrasentan, enalapril and paricalcitol on additional cardiovascular parameters is highly warranted.

## Figures and Tables

**Table 1:** Body Weight and Serum Chemistries

Group	Body Wt (g)	Cr (mg/dl)	ICa^++^ (mg/dl)	Total Ca (mg/dl)	P (mg/dl)	CaxP (mg^2^/dl^2^)
Nor (n=6)	263.7 ± 2.9	0.48 ±0.02	4.84 ± 0.04	9.96 ± 0.08	4.23 ±0.23	42.2 ± 2.3
UC (n=13)	259.2 ± 4.1	1.22 ± 0.11α	4.90 ± 0.04	11.17 ± 0.16α	5.84 ± 0.39α	65.5 + 5.0α
UE (n=14)	256.9 ± 2.6	0.87 ± 0.03α,β	4.91 ±0.04	10.82 ± 0.21β	4.81 ± 0.29	52.5 ± 3.9
UA (n=11)	274.6 ± 5.4	1.16 ±0.11α	4.95 ± 0.03	11.04 ±0.18α	5.75 ± 0.36α	63.8 ± 4.4α
UP (n=13)	256.2 ± 4.0	0.94 ± 0.06α	5.46 ± 0.07α,β	12.08± 0.23α,β	6.10±0.17α	73.8 ± 2.8α
UEAP (n=13)	266.7 ± 4.0	0.87 ± 0.05 α,β	5.29 ± 0.06α,β	11.43 ±0.17α	5.88 ±0.19α	67.3 ± 2.6α

Abbreviations: Ca, calcium; CaxP, calcium × phosphorus; Cr, creatinine; ICa^+ +^, ionized calcium; P, phosphorus; wt, weight. Rats were made uremic and treated as follows for 3 months: no treatment (UC), enalapril (UE), paricalcitol (UP), atrasentan (UA), or a combination of the 3 (UEAP). Normal rats served as controls (Nor).

α p≤0.05 *vs* Nor;

β p≤0.05 *vs* UC. Results are expressed as mean ± SEM.
